# Tetracysteine-Based Fluorescent Tags to Study Protein Localization and Trafficking in *Plasmodium falciparum*-Infected Erythrocytes

**DOI:** 10.1371/journal.pone.0022975

**Published:** 2011-08-10

**Authors:** Georgeta Crivat, Fuyuki Tokumasu, Juliana Martha Sa, Jeeseong Hwang, Thomas E. Wellems

**Affiliations:** 1 Laboratory of Malaria and Vector Research, Malaria Genetics Section, National Institute of Allergy and Infectious Diseases, National Institutes of Health, Bethesda, Maryland, United States of America; 2 Physical Measurement Laboratory, Biophysics Group, Optical Technology Division, National Institute of Standards and Technology, Gaithersburg, Maryland, United States of America; Kenya Medical Research Institute - Wellcome Trust Research Programme, Kenya

## Abstract

*Plasmodium falciparum (Pf)* malaria parasites remodel host erythrocytes by placing membranous structures in the host cell cytoplasm and inserting proteins into the surrounding erythrocyte membranes. Dynamic imaging techniques with high spatial and temporal resolutions are required to study the trafficking pathways of proteins and the time courses of their delivery to the host erythrocyte membrane.

**Methodology and Findings:**

Using a tetracysteine (TC) motif tag and TC-binding biarsenical fluorophores (BAFs) including fluorescein arsenical hairpin (FlAsH) and resorufin arsenical hairpin (ReAsH), we detected knob-associated histidine-rich protein (KAHRP) constructs in *Pf*-parasitized erythrocytes and compared their fluorescence signals to those of GFP (green fluorescent protein)-tagged KAHRP. Rigorous treatment with BAL (2, 3 dimercaptopropanol; British anti-Lewisite) was required to reduce high background due to nonspecific BAF interactions with endogenous cysteine-rich proteins. After this background reduction, similar patterns of fluorescence were obtained from the TC- and GFP-tagged proteins. The fluorescence from FlAsH and ReAsH-labeled protein bleached at faster rates than the fluorescence from GFP-labeled protein.

**Conclusion:**

While TC/BAF labeling to *Pf*-infected erythrocytes is presently limited by high background signals, it may offer a useful complement or alternative to GFP labeling methods. Our observations are in agreement with the currently-accepted model of KAHRP movement through the cytoplasm, including transient association of KAHRP with Maurer's clefts before its incorporation into knobs in the host erythrocyte membrane.

## Introduction

Upon invading erythrocytes, *Plasmodium falciparum (Pf)* parasites extensively remodel their host cells [1]. Each parasite surrounds itself with a parasitophorous vacuole membrane (PVM), which separates erythrocyte cytoplasm from the parasite-residing space. The absence of endogenous protein trafficking mechanisms in erythrocytes requires *Pf* parasites to install processes by which synthesized proteins can be transported to the surface of the host erythrocyte.

Some *Pf*-produced proteins are localized in electron-dense protrusions (“knobs”) at the parasitized erythrocyte (PE) surface [2], [3]. These proteins provide points of cytoadherence to endothelium of microvenules, enabling the parasitized erythrocytes to sequester and avoid elimination by the spleen [4], [5], [6], [7], [8], [9]. *Pf* erythrocyte membrane protein 1 (*Pf*EMP1) and knob-associated histidine-rich protein (KAHRP) are two important components of knobs [6], [7]. KAHRP serves as an essential structural element of knobs, binds to the membrane skeleton of the host erythrocyte, and helps to anchor the acid terminal segment (ATS) of *Pf*EMP1 [10], [11], which is responsible for cytoadherence through antigenically variant regions that recognize receptors such as CD36, TSP (thrombospondin), and ICAM-1 (Inter-Cellular Adhesion Molecule 1) [12], [13], [14]. A current model of intraerythrocytic protein delivery to knobs invokes parasite export of proteins across the PVM to the erythrocyte cytoplasm, transient association with Maurer's clefts and, finally, docking at the erythrocyte membrane [15], [16].

Since the original report of GFP for biological imaging [17], applications of this protein have greatly expanded to include different color variants with different emission spectra [18]. Previous work using GFP-tagged KAHRP, PfEMP1 and other proteins has identified amino acid sequences of protein export motifs, translocons at PVM and putative steps in the pathways by which constituent proteins of knobs reach the host erythrocyte membrane [19], [20]. Dynamic fluorescence techniques such as fluorescence recovery after photobleaching (FRAP) and Förster resonance energy transfer (FRET) can also be used to investigate intracellular trafficking and protein-protein interactions. FRAP has provided valuable insights on protein trafficking involving various compartments of the PE such as the parasitophorous vacuoles (PVs), PVM extensions, and erythrocyte cytoplasm [14]. However, the trafficking process may be perturbed by imperfect maturation of GFP fusion proteins, complications with oligomerization, or reduction of protein diffusion rates due to the non-negligible size of GFP (25 kDa to 27 kDa; 238 amino acids) [21], [22].

In recent years, other alternative labeling techniques have also been developed, including the tagging of target proteins with short peptides that carry a six amino acid tetracysteine (TC) motif, -Cys-Cys-Xaa-Xaa-Cys-Cys- (where Xaa is an amino acid other than Cys) [23]. This six amino acid tag binds membrane-permeable biarsenical fluorophores (BAFs) such as FlAsH and ReAsH [24]. Before binding to the TC tag, FlAsH and ReAsH are nonfluorescent in the form of FlAsH-EDT_2_ or ReAsH-EDT_2_, where EDT (1,2-ethanedithiol) moieties quench the fluorescence by vibrational deactivation. Upon BAF binding to the TC motif, the EDT is displaced and BAF becomes fluorescent [24]. Because BAFs are highly membrane permeable, unbound molecules within the cell can be washed away. These unique properties of the BAF have enabled two-color pulse-pulse labeling and time series studies of protein–protein interactions, protein synthesis and trafficking in live cells [25], [26], [27], [28]. In a typical two-color application, two BAFs with distinct emission spectra are applied to TC-tagged proteins to image and track proteins produced at different times. In contrast, in GFP-based imaging techniques such imaging and tracking is available only with a photoactivatable form of GFP [29]. Another potential advantage of TC-tagging is the ability of ReAsH to support the photoconversion of diaminobenzidine (DAB) into osmiophilic residues, which can be observed by electron microscopy [25]. Finally, TC tags are short so they are less likely than GFP tags to interfere with protein expression or to affect protein structure; like GFP tags, TC tags do not require antibody binding for imaging.

Here we report on a study of the potential applicability and limitations of FlAsH and ReAsH labels bound to TC-tagged KAHRPs in PEs. The fluorescence images from this study are in agreement with a leading model of KAHRP trafficking to the host erythrocyte membrane.

## Materials and Methods

### DNA constructs

**Figure 1:**
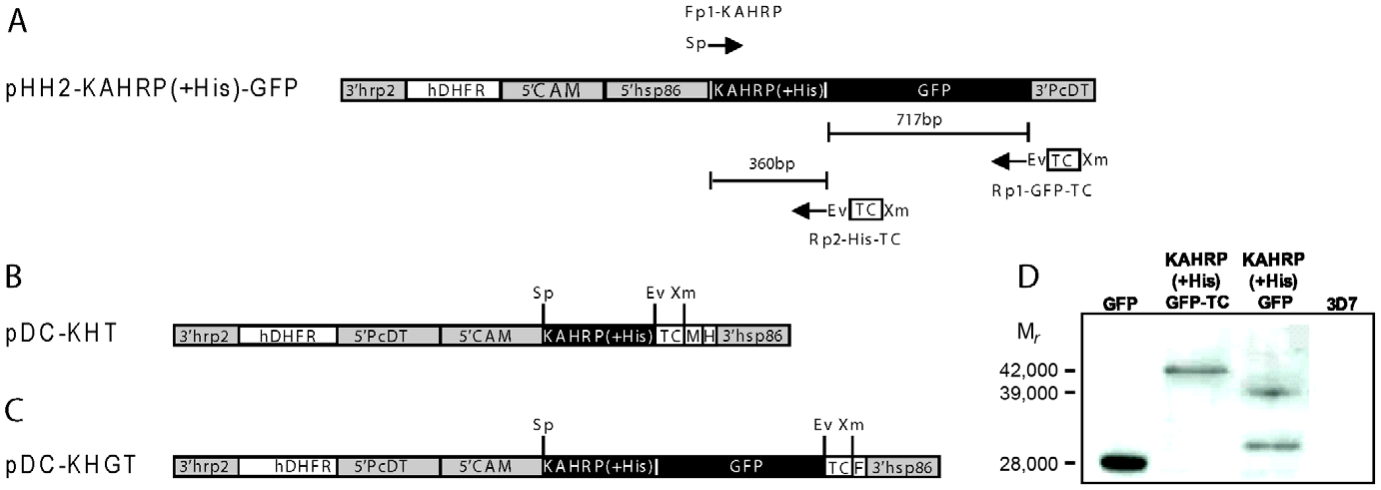
Transfection plasmids and detection of GFP fusion proteins. (A) Plasmid pHH2-KAHRP(+His)-GFP [14] was used to amplify the first 60 amino acids of KAHRP containing the putative hydrophobic signal sequence followed by the histidine rich region with or without GFP fusion. *3′hrp2*, histidine-rich protein-2 3′UTR; *hDHFR*, human dihydrofolate reductase gene; *5′CAM*, *Pf* calmodulin promoter region; *5′hsp86*, heat-shock protein-86 promoter region; *3′PcDT*, *P. chabaudi* dihydrofolate reductase 3′UTR; Fp1-KAHRP, forward primer used to add a *Spe*I restriction site (Sp) immediately before the first codon of KAHRP cloned into pDC-pvcrt-o-MH (not shown); Rp1-GFP-TC, reverse primer used to amplify GFP, add an *Eco*RV restriction site (Ev) in frame between GFP and the TC tag (for subcloning of other genes), and add a *Xma*I restriction site (Xm) after the TC sequence, for cloning into pDC-pvcrt-o-MH; Rp2-His-TC, reverse primer used to amplify the histidine rich region of KAHRP, add an *Eco*RV restriction site (Ev) while maintaining the reading frame of KAHRP(+His) and the TC tag (for future subcloning of other genes), and add a *Xma*I restriction site (Xm) after the TC sequence, for cloning into pDC-pvcrt-o-MH. (B) Transfection plasmid pDC-KHT contains the sequence for the fusion protein KAHRP(+His)-TC from pHH2-KARHP(+His)-TC. Myc (M) and His×6 (H) tags are encoded immediately after the *Xma*I restriction site. (C) Transfection plasmid pDC-KHGT contains the sequence of KAHRP(+His)-GFP-TC in frame with codons for a PRGTKTYF terminus (F) that begin at the *Xma*I site. *5′PcDT*, *P. chabaudi* dihydrofolate reductase promoter region; *3′hsp86*, heat-shock protein-86 3′UTR. (D) Immunoblot image shows antibody detection of GFP as a M*_r_* 28,000 band, detection of KAHRP(+His)-GFP-TC as a M*_r_* 42,000 band, and detection of M*_r_* 39,000 and M*_r_* 29,000 bands from KAHRP(+His)-GFP protein. No band was detected from control non-transformed 3D7 PE.

Plasmid pHH2-KAHRP(+His)-GFP (Fig. 1A, [14]), kindly provided by Dr. Alan Cowman, was used as a template to PCR amplify the N-terminal sequence of KAHRP, its histidine-rich region, and GFP. Plasmid pDC-pvcrt-o-MH (a modified version of plasmid pDC-CAT that includes 16 codons for adjoining Myc and His×6 tags at the C′ terminus of *pvcrt-o* or other gene of interest [30]; not shown) was used to directly clone the amplified coding sequences of KAHRP(+His)-TC or KAHRP(+His)-GFP-TC between *Spe*I and *Xma*I restrictions sites. We designed a single forward oligonucleotide primer containing a *Spe*I restriction site followed by the N-terminal region of KAHRP: Fp1-KAHRP 5′ ggactagtATGAAAAGTTTTAAGAACAAAAATACTTTGAGGAGAAAGAAGGCTTTCCC 3′ (*Spe*I restriction site is underlined). Reverse oligonucleotide primers were designed to include coding sequences of the TC-containing motif (amino acid sequence RTGAGG**CCPGCC**GGG; TC residues underlined) and the C-terminal sequence of either KAHRP(+His) alone or of KAHRP(+His)-GFP; these reverse primers were designed without stop codons, to allow a potentially continuous open reading frame with the Myc and His×6 tags encoded by the pDC-pvcrt-o-MH plasmid. To facilitate the future cloning of other genes of interest followed by the TC motif in our transfection plasmids, we also included a six-nucleotide *Eco*RV restriction site in these reverse oligonucleotide primers, which maintains the reading frame of the TC motif: Rp1-GFP-TC 5′-tccccccggg **GCCGCCACCGCAACAGCCAGGACAACAGCCACCAGCACCGGTACG** *gatatc*TTTGTATAGTTCATCCATGCCATGTGTAATCCCAGCAGC -3′ and Rp2-His-TC 5′-tccccccggg **GCCGCCACCGCAACAGCCAGGACAACAGCCACCAGCACCGGTACG** *gatatc*AGGTTGTAATTGATGATGGTGGTGATGATGGTGATGGTG-3′ (the *Xma*I restriction site for cloning into pDC-pvcrt-o-MH is in lower case and underlined; the *Eco*RV restriction site is italicized and underlined; the 15 codons of the TC tag are shown in bold upper case with the CCPGCC codons underlined). Fragments KAHRP(+His)-GFP-TC and KAHRP(+His)-TC were amplified from pHH2-KAHRP(+His)-GFP with primers Fp1-KAHRP plus Rp1-GFP-TC and with Fp1-KAHRP plus Rp2-KAHRP-TC, respectively, using 30 repetitions of the following PCR-thermo cycle: 30 s denaturation at 94°C; 60 s annealing at 60°C (for KAHRP(+His)-GFP-TC) or at 65°C (for KAHRP(+His)-TC); and 120 s extension at 68°C. The purified fragments were cloned into pGEM-T Easy vector (Stratagene, Santa Clara, CA), confirmed by sequencing (Clinical Laboratory Improvements Amendments Molecular Diagnostics & Sanger Sequencing Group, Frederick, MD), digested with *Spe*I and *Xma*I, and ligated into pDC-pvcrt-o-MH which had also been digested with *Spe*I and *Xma*I. The resulting plasmids, pDC-KHGT and pDC-KHT, were checked for integrity and orientation of the inserts by restriction analysis and DNA sequencing. Results showed that plasmid pDC-KHT contains the sequence of KAHRP(+His)-TC in frame with the codons of the Myc and His×6 tags, as expected (Fig. 1B). Plasmid pDC-KHGT, however, contains an inserted extra cytosine immediately upstream the *Xma*I restriction site; this extra cytosine does not affect the codons of the KAHRP(+His)-GFP-TC sequence but it places the downstream Myc and His×6 tag codons out of frame, so that the encoded protein terminates instead with the amino acid sequence PRGTKTYF (Fig. 1C). Because this extra cytosine did not affect the GFP and TC sequences of the pDC-KHGT plasmid for control experiments, it was not removed for these studies. The sequences of the pDC-KHT and pDC-KHGT plasmids are deposited in GenBank (accession numbers: JF430587 and JF430586).

### Parasite culture and parasite transformation

*Pf* 3D7 parasite cultures were maintained between 1% to 5% parasitemia in RPMI complete media which contains RPMI 1640+GlutaMAX™ (Invitrogen, Carlsbad, CA) supplemented with 5 mg/mL AlbuMAX® (GIBCO, Invitrogen) and 20 µg/mL gentamicin, under an atmosphere of 5% O_2_/5% CO_2_/90% N_2_ (v/v/v, Roberts Oxygen, Rockville, MD) at 37°C. To insure that the 3D7 population did not lose expression of knobs, PE were periodically subjected to selection by gelatin flotation [31]. Loading of plasmid DNA into uninfected erythrocytes by electroporation was performed as previously described [32]. Gelatin-floated PE were added to the electroporated erythrocytes and, after spontaneous uptake of plasmid DNA from the host erythrocyte cytoplasm, transformed PE were selected with 5 nmol/L of the antifolate drug WR99210 (4,6-diamino-1,2-dihydro-2,2-dimethyl-1-[(2,4,5-trichlorophenoxy)propyloxy]-1,3,5-triazine). From these transfections, we obtained the following three *Pf* 3D7 transformed lines with the associated GFP and/or TC tags: 3D7-KAHRP(+His)-TC (containing plasmid pDC-KHT DNA); 3D7-KAHRP(+His)-GFP-TC (containing plasmid pDC-KHGT DNA); and the control line 3D7-KAHRP(+His)-GFP (containing plasmid pHH2-KAHRP(+His)-GFP DNA [14]).

### Protein analysis by immunoblotting

Mature parasitized erythrocytes were isolated for immunoblotting on LS magnetic separation columns (Miltenyi Biotec, Auburn, CA) as described elsewhere [33]. From the column, approximately 5×10^6^–10×10^6^ mature PE were obtained in 2 mL RPMI complete media, centrifuged at 2,200 rpm for 5 min, and washed with a phosphate buffered saline (PBS, Na_2_HPO_4_ 795 mg/L; KH_2_PO_4_ 144 mg/L; NaCl 9000 mg/L; pH 7.4) solution. Parasitized cells were freed of hemoglobin by treatment with 150 mg/mL saponin in a total reaction volume of 500 µL PBS for 10 min on ice. After centrifugation at 13,000 rpm for 5 min, the pellet was recovered, resuspended in PBS, pelleted again, and resuspended in 1% triton X-100 in ice-cold PBS in the presence of serine and cysteine protease inhibitors according to manufacturer protocols (Roche Diagnostics, Mannheim, Germany). Samples were combined with NuPAGE lithium dodecyl sulfate (LDS) sample buffer and NuPAGE reducing agent (Invitrogen), heated for 5 min at 70°C, fractionated in a bis-Tris 4% to 12% polyacrylamide gel (Invitrogen), and transferred to a polyvinylidene fluoride (PVDF) membrane as recommended by the supplier (Invitrogen). After overnight treatment in Sigma blocking buffer (Sigma-Aldrich, St. Louis, MO), the membrane was incubated with anti-GFP mouse IgG monoclonal antibody (Roche Diagnostics, Indianapolis, IN) at 1∶5,000 dilution in blocking buffer for 2 h at room temperature. The membrane was washed with 0.2% Tween-20 solution in PBS, and incubated with horseradish peroxidase (HRP)-conjugated goat anti-mouse IgG antibody (Jackson ImmunoResearch Laboratories, West Grove, PA) diluted 30,000 times in blocking buffer for 1 h. Bands were developed with Super Signal® West Pico chemical luminescence solutions (Pierce, Thermo-Fisher Scientific, Rockford, IL) and Hyperfilm ECL paper (GE Healthcare, Piscataway, NJ). Purified recombinant GFP (Roche) was used as positive control.

### Wide-field fluorescence light microscopy

Wide-field fluorescence images were recorded using a 100× (N.A. 1.4) objective in a Leica DMI 6000B inverted optical microscope (Leica Microsystems, Bannockburn, IL). Micrographs were acquired using a Hamamatsu Orca ER digital CCD camera (Hamamatsu Photonics System, Bridgewater, NJ) and Image Pro image acquisition software (Mediacybernetics, Bethesda, MD). Green fluorescence from GFP and FlAsH bound to TC-tagged proteins was recorded using an XF100-2 fluorescence filter set (excitation 475AF40; emission 535AF45; Omega Optical, Brattleboro, VT); red fluorescence from ReAsH bound to TC-tagged proteins was recorded using an XF102-2 set (excitation 560AF55; emission 645AF75; Omega Optical, Brattleboro, VT). The excitation power for each optical filter set was measured with a wavelength-corrected, calibrated power meter (S120C, Thorlabs Inc., NJ) by placing a silicon photodiode detector head at the focal plane of the objective lens. The scale of the intensity adjustment knob for field diaphragm control was calibrated against the power measurement.

### Photobleaching comparisons of GFP vs. BAFs

Fluorescence photobleaching was measured on live cells under continuous exposure to focused light at the excitation wavelength. Images were captured at a frame rate of 3 frames/s for 20 s at 9.98 mW of incident light through a 100× objective for GFP and FlAsH, and at 10.88 mW for ReAsH. No anti bleaching reagents were added to the media.

### Reduction of nonspecific binding background of BAFs

Although Griffin et al. [24] estimated a low binding of the endogenous cysteine-rich regions of cellular proteins to BAFs, other reports showed different levels of nonspecific background staining, which could result in poor signal-to-noise ratios (S/N) depending on the type of cell line and target protein [34], [35]. Methods to overcome the nonspecific background have included the use of BAL (2,3 dimercaptopropanol; British anti-Lewisite) or EDT (ethanedithiols) to reduce the affinity of BAFs for endogenous peptides containing CCXXCC or CXXC [23], [36], [37] or other Cys-rich amino acid sequences.

Our initial images of 3D7 non-transformed parasites exposed to FlAsH or ReAsH showed unacceptable background fluorescence. To reduce nonspecific background, the cells were pre-treated with 650 µmol/L BAL in incomplete RPMI 1640 (without serum, albumin and phenol-red, RPMI-inc) for 15 min at 37°C, followed by washes with RPMI-inc at room temperature. Use of RPMI-inc during labeling and washing avoided unnecessary background effects from the interaction of albumin with BAFs [37].

The BAL-treated samples were then exposed to 2.5 µmol/L BAFs for 30 min at 37°C. ReAsH labeling was followed by 3 consecutive washes with 250 µmol/L BAL in warm RPMI-inc to remove ReAsH-BAL complexes and unbound or loosely bound ReAsH. An additional treatment with 250 µmol/L BAL in RPMI-inc containing 20 µmol/L Disperse Blue (Sigma) for 10 min to 40 min at 37°C was added to displace ReAsH that was tightly bound to nonspecific sites by dithiol-independent hydrophobic interactions [37]. After a final wash with warm RPMI-inc only, the samples were examined by fluorescence microscopy.

FlAsH labeling was followed by 3 washes with 250 µmol/L BAL in warm RPMI-inc; 15 min treatment at 37°C with 250 µmol/L BAL in RPMI containing 20 µmol/L Disperse Blue; 15 min treatment at 37°C with 500 µmol/L BAL in RPMI-inc containing 20 µmol/L Disperse Blue; and a final wash with warm RPMI-inc only.

S/N ratios of ReAsH and FlAsH were calculated using the following equation: (F_specific_−F_nonspecific_)/F_nonspecific_; where F_specific_ represents the average pixel fluorescence intensity from the BAF in transgenic PE expressing the TC tag, and F_nonspecific_ is the average pixel fluorescence intensity of cells with non- transformed 3D7 parasites treated with the same BAF.

To assess endogenous cysteine-rich proteins as a possible source of nonspecific background in the non-transformed 3D7 parental line, we pretreated 3D7 PE with 200 µmol/L coumarin maleimide (CPM) in RPMI-inc for 1 h at 37°C followed by labeling with 2.5 µmol/L ReAsH. CPM excitation and emission were 380 nm and 407 nm, respectively. CPM is highly reactive to thiol groups, exhibits no fluorescence in an unbound state, and has been used to block endogenous cysteines [34]. Analysis of fluorescence intensities was performed using Image Pro image software.

### Effect of BAL on Pf in vitro growth

To determine whether BAL would have an adverse effect on the parasite growth after the 15 min BAL pre-treatment for background reduction, we performed *in vitro* growth inhibition assays with various concentrations of BAL and at two incubation times, 15 min and 1 h. A modified protocol from the SYBR Green cell multiplication detection method established by Smilkstein et al. [38] was used. Synchronized ring stage cultures at 1% parasitemia were incubated for 15 min at 37°C at BAL concentrations of 0 mmol/L to 5 mmol/L and then washed with RPMI-inc. The PE were then resuspended in complete RPMI and cultured for 72 h. Parasite growth was measured by DNA multiplication detected by SYBR Green.

## Results

### Expression of KAHRP (+His)-GFP-TC

We constructed two transfection plasmids from the original plasmid that expresses KAHRP(+His)-GFP (pHH2-KAHRP(+His)-GFP) [14]: plasmid pDC-KHT, which encodes KAHRP(+His) followed by TC, Myc and His×6 tags but not GFP (Fig. 1B); and pDC-KHGT, which encodes KAHRP(+His) followed by GFP, the TC tag, and a PRGTKTYF terminus that resulted from a single nucleotide frame shift at the *Xma*I site (Fig. 1C). The KAHRP(+His) domain in these constructs provides a histidine-rich region necessary for protein delivery to erythrocyte surface [14]. To confirm expression of intact fusion protein from plasmid pDC-KHGT, we used anti-GFP antibodies to probe immunoblots of the transformed parasite line 3D7-KAHRP(+His)-GFP-TC (Fig. 1D). Positive controls included purified recombinant GFP protein and extracts of the transformed line 3D7-KAHRP(+His)-GFP (containing plasmid pHH2-KAHRP(+His)-GFP DNA); negative control was an extract of non-transformed 3D7 parasites. In the positive control, purified recombinant GFP (predicted molecular mass of 27 kDa) was detected as a single band with relative molecular weight (*M_r_*) of 28,000 (Fig. 1D), while two bands with *M_r_*≈29,000 and *M_r_*≈39,000 were detected from 3D7-KAHRP(+His)-GFP parasites. These two bands from transformed 3D7-KAHRP(+His)-GFP parasites are in agreement with the original report by Wickham *et al.* [14], in which KAHRP(+His)-GFP has a predicted molecular mass of 37.6 kDa due to possible cleavage at the predicted Cys34-Ser35 site. The slightly greater M*_r_* on our immunoblots likely reflects altered electrophoretic mobility due to the histidine-rich nature of KAHRP.GFP fusion proteins have been reported to show an additional band of *M_r_*≈29,000 corresponding to a GFP-cleavage product [14], [39]. The expressed protein from our pDC-KHGT-transformed 3D7 parasites shows a 42,000 *M_r_* band, consistent with the addition of the TC tag and PRGTKTYF terminus (together approximately 2.7 kDa). Absence of a detectable cleavage band on the 3D7-KAHRP (+His)-GFP-TC immunoblot may be due to an effect of the TC tag and/or the PRGTKTYF terminus on susceptibility of the protein to proteolysis.

### Optimization of FlAsH and ReAsH fluorescence labeling

**Figure 2:**
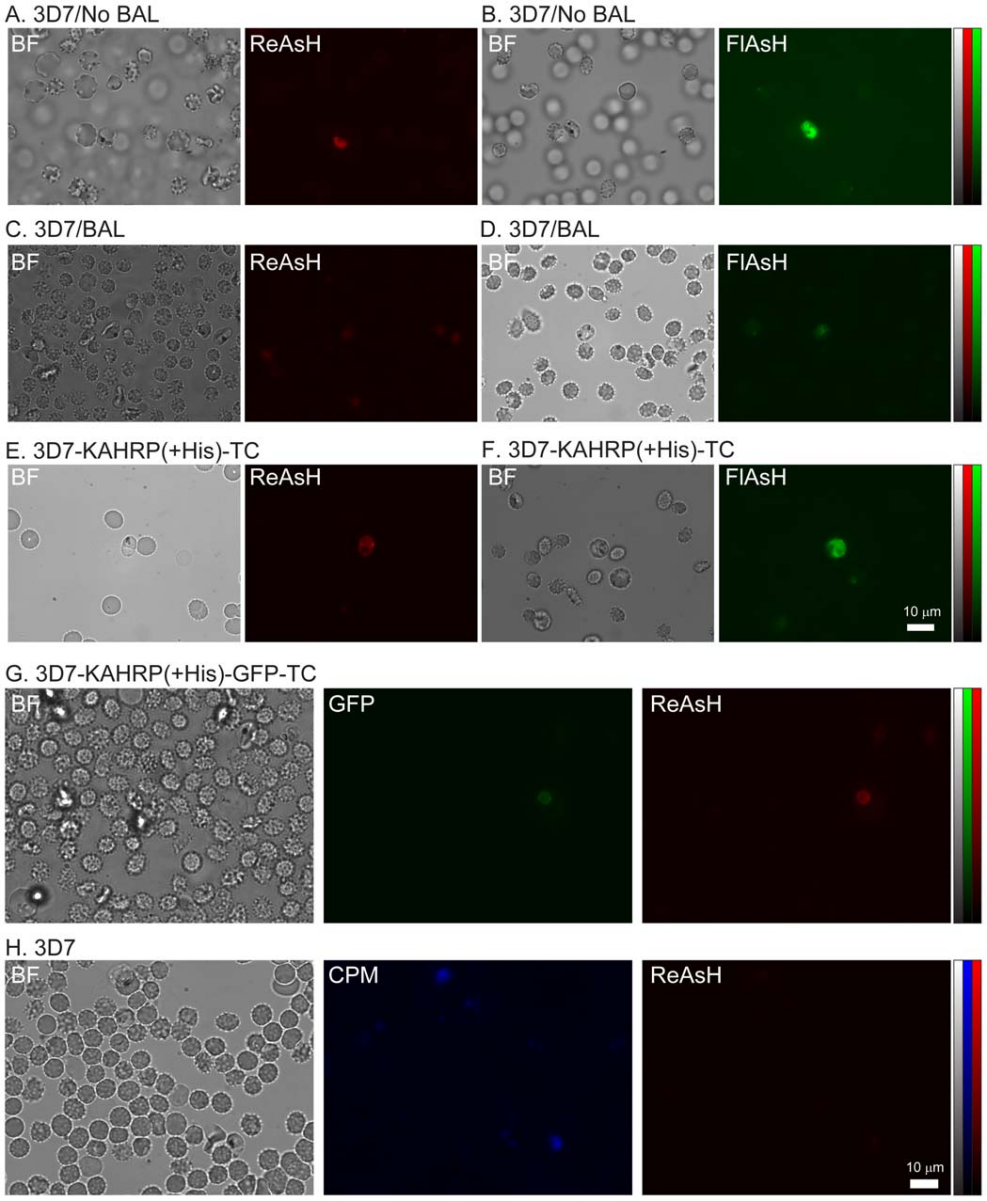
Fluorescence microscopy images of ReAsH and FlAsH labeled PEs. (A) Bright field (BF) and fluorescence images of non-transformed 3D7 PE labeled with ReAsH, not treated with BAL. (B) BF and fluorescence images of non- transformed 3D7 PE labeled with FlAsH, not treated with BAL. (C) BF and fluorescence images of 3D7 PE treated with BAL and labeled with ReAsH. (D) BF and fluorescence images of 3D7 PE treated with BAL and labeled with FlAsH. (E) BF and fluorescence images of 3D7-KAHRP(+His)-TC PE treated with BAL and labeled with ReAsH. (F) BF and fluorescence images of 3D7-KAHRP(+His)-TC PE treated with BAL and labeled with FlAsH. (G) BF and fluorescence images of 3D7-KAHRP(+His)-GFP-TC PE treated with BAL and labeled with ReAsH. (H) Images of non- transformed 3D7 PE exposed first to 200 µmol/L CPM and followed by labeling with 2.5 µmol/L ReAsH. Excitation and imaging of ReAsH, FlAsH and CPM fluorescence were performed with wavelengths and filter sets described in Materials and Methods. Scale bar represents 10 µm.

In pilot experiments we found that background fluorescence intensity from non- transformed parasites in PEs treated directly with FlAsH or ReAsH was unacceptably high (Figure 2A and 2B). Therefore, various treatments of the PEs with 650 µmol/L BAL before BAF labeling and washes with 250 to 500 µmol/L BAL+20 µmol/L Disperse Blue after BAF labeling were explored.

With non-transformed 3D7 parasites treated with ReAsH and FlAsH, we were able to achieve markedly reduced but still detectable nonspecific background with a 650 µmol/L BAL pre-treatment (prior to BAF labeling) followed by three consecutive washes with 250 µmol/L BAL and a further wash with either 250 µmol/L BAL (ReAsH) containing 20 µmol/L Disperse Blue or 500 µmol/L BAL containing 20 µmol/L Disperse Blue (FlAsH) (Fig. 2. C, D). Further experiments showed that the pre-treatment BAL concentration could be lowered to 100 µmol/L without greatly increasing the background, but 50 to 100 µmol/L BAL in the post-labeling washes was not enough to adequately control the background fluorescence. Little or no nonspecific background was evident in non-parasitized erythrocytes labeled with BAFs after BAL treatment.

Transformed 3D7-KAHRP(+His)-TC parasites treated with ReAsH showed good retention of positive signal after treatment to reduce background (Fig. 2.E); slightly higher signals were detected from parasites treated with FlAsH (Fig. 2.F). However, some cell-to-cell variations in the emission intensity within the same culture were noticed, probably due to age differences or infections with multiple parasites. In addition, we observed that BAL treatment not only reduces the unspecific background, but also the overall positive signal.

Fluorescence images of dual GFP- and ReAsH-labeled 3D7-KAHRP(+His)-GFP-TC parasites showed a comparable retention and distribution of fluorescence signal after applying the procedures for background reduction (Fig. 2G).

These results agree with the calculated S/N ratio (Table 1). Among TC-expressing parasites treated with BAFs, we found a lower S/N ratio for FlAsH in 3D7-KAHRP(+His)-TC parasites than in ReAsH treated PEs. FlAsH has higher affinity than ReAsH for specific [23] and nonspecific sites consistent with a lower S/N than for ReAsH (compare Fig. 2 F&D vs. Fig. 2 E&C). GFP signals from 3D7-KAHRP(+His)-GFP-TC PEs provided the highest S/N ratio as no non-specific background binding of a GFP fluorophore is involved.

**Table 1:** S/N ratios of GFP, FlAsH and ReAsH fluorescence from 3D7-KAHRP(+His)-GFP-TC- and 3D7-KAHRP(+His)-TC-transformed PE.

Parasite	Signal/Noise		
Line	GFP	FlAsH	ReAsH
3D7-KAHRP(+His)-GFP-TC	3.25±1.77	–	2.65±1.71
3D7-KAHRP(+His)-TC	–	1.23±0.85	3.02±2.29

We observed crenation of erythrocytes due to possible environmental changes (chemicals, temperature, osmotic pressure) during labeling, treatment to reduce background, or the image recording process. However, these morphological changes were reversed by returning erythrocytes to the culture condition [40], [41].

It was suggested that cysteine-rich proteins generally have higher affinity to BAFs [34]. To test whether cysteine-rich molecules give rise to the nonspecific background in *Pf* parasites, we treated non-transformed 3D7 cells with thiol-reactive UV excitable CPM. We observed a high blue fluorescence from parasitized erythrocytes, suggesting the presence of abundant endogenous cysteines and other thiol-containing molecules inside PE (Fig. 2H). After CPM treatment, labeling with ReAsH did not result in significant red background fluorescence (compare Fig. 2H).

As previously reported [37], we observed that dying or dead cells (including non-transformed 3D7 parasites) fluoresced brightly and remained particularly stubborn in their fluorescence during background removal. These cells were excluded from data analysis.

### BAL toxicity assessments

Because of the high levels of BAL treatment required for reduction of background, the effect of BAL on the growth and development of live parasites was tested. In these experiments non-transformed 3D7 and transformed 3D7-KAHRP(+His)-GFP-TC, and 3D7-KAHRP(+His)-TC PE were exposed to serially diluted concentrations of BAL for 15 min or 1 h and then cultured without BAL for 72 h. SYBR green assays after the 15 min exposures yielded IC_50_ values of 1.65 mmol/L±0.33 mmol/L for 3D7 and 1.25 mmol/L±0.31 mmol/L for 3D7-KAHRP(+His)-TC parasites (Table 2). These IC_50_ values are above the highest concentration of BAL used in background reduction treatment (0.650 mmol/L). However, 60 min exposure to BAL yielded IC_50_ values below 0.650 mmol/L: 0.50 mmol/L±0.08 mmol/L for 3D7 and 0.38 mmol/L±0.02 mmol/L for 3D7-KAHRP(+His)-TC parasites (Table 2). Generally lower IC_50_'s were observed for 3D7-KAHRP(+His)-GFP-TC parasites relative to 3D7-KAHRP(+His)-TC or non-transformed 3D7 parasites.

**Table 2:** Measurements of cell multiplication and 50% and 90% inhibitory concentrations (IC_50_; IC_90_).

Parasite	15 minutes exposure to BAL						1 hour exposure to BAL					
Line	IC_50_ (mmol/L)			IC_90_ (mmol/L)			IC_50_ (mmol/L)			IC_90_ (mmol/L)		
3D7	1.65	±	0.33	4.43	±	0.86	0.50	±	0.08	1.37	±	0.33
3D7-KAHRP(+His)-GFP-TC	0.78	±	0.07	2.60	±	0.38	0.33	±	0.01	0.65	±	0.08
3D7-KAHRP(+His)-TC	1.25	±	0.31	2.88	±	0.72	0.38	±	0.02	0.90	±	0.14

Values are shown as mean of 3 experiments in duplicate with standard errors. Parasites lines 3D7, 3D7-KAHRP (+His)-GFP-TC and 3D7-KAHRP (+His)-TC were exposed for 15 min, and 1 h to different concentrations of BAL (0 mmol/L–5 mmol/L).

### Fluorescence patterns from TC-tagged KAHRPs in parasitized erythrocytes

**Figure 3:**
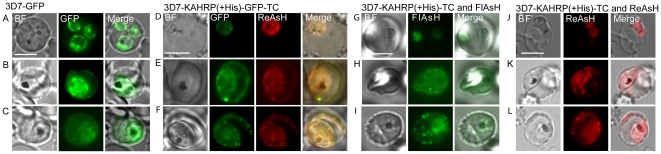
KAHRP protein trafficking in PEs. (A–C) Bright field (BF) and GFP fluorescence images of PEs containing *Pf* 3D7 parasites transformed to express KAHRP(+His)-GFP protein without TC tag. (D–F) Images of 3D7-KAHRP(+His)-GFP-TC PE labeled with ReAsH and photographed in the green and red channels separately for GFP and ReAsH fluorescence. (G–I) Images of 3D7-KAHRP(+His)-TC PE labeled with FlAsH. (J–L) Images of 3D7-KAHRP(+His)-TC PE labeled with ReAsH. The emission crossover between green and red channels was negligible, and was estimated to be less than a 1% leak of GFP emission into the ReAsH emission channel. Scale bar represents 5 µm.

To demonstrate the applicability of TC-tagged proteins in the study of trafficking behaviors of *Pf*-produced KAHRPs, we compared fluorescence patterns from the KAHRP(+His)-GFP protein described by Wickham *et al.* [14] (Fig. 3A–C), KAHRP(+His)-GFP-TC protein labeled with ReAsH (Fig. 3D–F), and KAHRP(+His)-TC protein labeled with FlAsH or ReAsH (Fig. 3G–L) in PEs containing various stages of transformed 3D7 parasites. In all cases, the observed patterns were comparable to those described by Wickham et al. [14]. Further, images from ReAsH-labeled 3D7-KAHRP(+His)-GFP-TC parasites (Fig. 3D–F) confirmed that the GFP and ReAsH fluorescence patterns co-localized as expected for ReAsH binding to TC adjacent to the GFP tag; and similar protein distributions were observed for FlAsH- or ReAsH-labeled 3D7-KAHRP(+His)-TC protein in PEs containing rings (Fig. 3A, D, G, J) and early and late trophozoites (Fig. 3B–C, E–F, H–I, K–L). Fluorescence from the tagged KAHRP first appeared within ring stage parasites and was associated with the PVM (Fig. 3A, D, G, J). Progression to trophozoites was accompanied by the appearance of fluorescent spots in the host erythrocyte cytoplasm often near the host membrane (Fig. 3B, E, H, K). These fluorescent concentrates of protein have been shown to be associated with Maurer's clefts [14]. In later trophozoite stages, concentrates of protein and an increased component of fluorescence were associated with the erythrocyte membrane (Fig. 3C, F, I, L).

### Photobleaching characteristics of BAF-TC vs. GFP tags in PE

**Figure 4:**
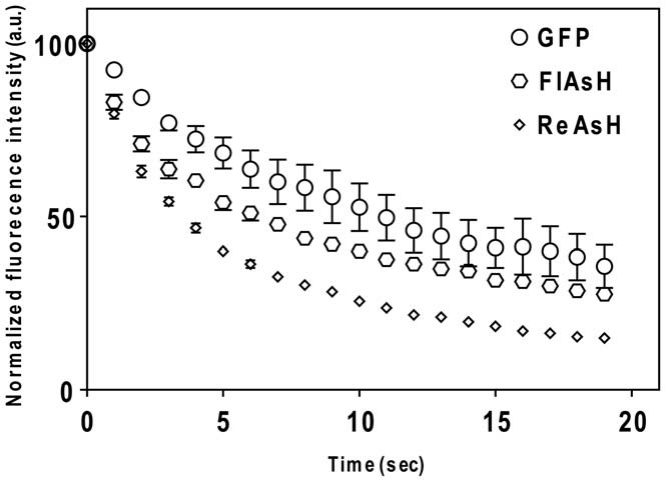
Photobleaching decay curves of GFP from 3D7-KAHRP(+His)-GFP-TC parasites, and ReAsH and FlAsH from 3D7-KAHRP(+His)-TC labeled parasites in *Pf-*infected erythrocytes. Continuous exposure of cells for 20 s to the 9.98 mW 475 nm±20 nm light (for GFP and FlAsH), or to the 10.88 mW 560 nm±22 nm light (for ReAsH) cause fluorescence decays to 65%, 73%, and 85% of the initial emission signals from GFP, FlAsH-, and ReAsH-labeled TC tagged protein, respectively.

Fluorescence decay rate is an important consideration for both qualitative and quantitative fluorescence imaging. In comparisons of the photobleaching characteristics of GFP in 3D7-KAHRP(+His)-GFP-TC PEs and FlAsH in 3D7- KAHRP(+His)-TC PEs, excitation with 9.98 mW of 475 nm±20 nm light for 20 s reduced the fluorescence intensities to 65% and 73% of their initial values, respectively. In other comparisons, ReAsH-labeled 3D7-KAHRP(+His)-TC parasites consistently bleached at the fastest rates, resulting in 85% decrease of the initial emission signals (Fig. 4). Although the BAF-TC tags exhibit faster photobleaching rates than GFP tags, only a small percentage of BAF fluorescence was bleached away during a typical image acquisition period (less than 500 ms), providing sufficient photostability for a study of protein trafficking in PE.

## Discussion

TC-tag labels and BAF offer fluorescence detection complementary to GFP for the study of protein trafficking in PEs. Our results show that ReAsH labeling of KAHRP protein tagged with both TC and GFP yielded similar fluorescence patterns at red and green wavelengths. Similar patterns were also obtained after FlAsH or ReAsH labeling of KAHRP tagged with TC alone, supporting previous assumptions that the larger GFP tag does not detectably interfere with this protein trafficking [14]. The limiting factor of this technique in malaria PEs is the high background from BAF labeling, consequently interfering with the application of FlAsH-TC or ReAsH-TC to two-color pulse labeling applications.

A major source of nonspecific background fluorescence with BAFs is their binding to CXXC motifs present, for example, in zinc finger proteins, RING finger proteins, protein kinases and cytoskeletal proteins. Coulson et al. [42] found that CCCH-type zinc finger motifs were encoded abundantly in the *Pf* genome. Additionally, PfCRMP1 and PfCRPM2 *Pf* Cysteine Repeat Modular Domain 1 and 2) [43] include CXXC motifs, which may also compound the high Cys-nonspecific background in *Pf* infected erythrocytes. Another source for background is non-thiol binding sites of hydrophobic pockets in endogenous proteins [34]. We were able to partially but not completely reduce nonspecific thiol-dependent background by using the thiol-containing reagent BAL. As previously reported, pre-treatment with CPM completely blocked background staining [34], but no red fluorescence was evident after subsequent ReAsH labeling (Fig. 2H) confirming that thiol-dependent BAF binding is the major source of background in *Pf* parasites.

BAL, an antidote to arsenic poisoning [44], is reported to reduce background more efficaciously and with less toxicity to cells than EDT [23], [45], [46]. To assess BAL toxicity, we determined the IC_50_ and IC_90_ for the different *Pf* lines for periods comparable to the BAL treatment times. The IC_50_ values of 3D7-KAHRP(+His)-TC-transformed parasites were above the concentration of BAL treatment prior to BAF labeling (650 µmol/L), and the red and green fluorescence patterns of ReAsH-labeled 3D7-KAHRP(+His)-GFP-TC-transformed parasites were comparable to those reported for KAHRP(+His)-GFP transformed parasites [14], suggesting that sub-cellular protein localization was not noticeably disturbed by the BAL treatment for background reduction. The slightly lower IC_50_s of GFP-expressing PE relative to PE not expressing GFP could be due to effects of apoptosis induced by GFP [47].

In future studies it might be possible to improve signal strength over background by engineering tandem TC motifs, as approached in other systems [48]. The use of a stronger promoter for higher exogenous protein expression may also enhance the signal from BAF labeled TC tags. The binding affinity of FlAsH for TC has been reported to be higher than that of ReAsH [23]; in our study, a lower S/N ratio for FlAsH relative to ReAsH suggests that non-specific binding of FlAsH is also greater than that of ReAsH.

A widely accepted model of KAHRP, *Pf*EMP1 and *Pf*EMP3 trafficking to the erythrocyte membrane involves movement of these proteins across the erythrocyte cytoplasm to Maurer's clefts followed by a step in which these proteins are transferred (probably as complexes) to knobs in the host erythrocyte membrane [7], [14], [49]. Maurer's clefts may be involved in the sorting and arrangement of these proteins before they are incorporated into the erythrocyte membrane [7], [16], [49], [50], but the details of the movement from Maurer's clefts to the erythrocyte plasma membrane still remain unclear. Recent electron tomography studies suggest that nascent Maurer's clefts from PVM interact with the cytoplasmic side of the PE membrane through stalk-like extensions of the clefts [15]. Vesicle-like structures attached to Maurer's clefts or to the erythrocyte membrane have also been observed. The roles of these structures in protein trafficking remain to be clarified [49].

In summary, our results from BAF-labeled TC tags are consistent with KAHRP trafficking through the cytoplasm and accumulation at Maurer's clefts prior to transfer to the host PE membrane, in agreement with the trafficking model proposed by Wickman *et al.* and Tilley *et al.* [14], [49]. Fluorescence from ring stage transgenic parasites expressing GFP-TC- or TC-tagged KAHRP proteins was within the PV; fluorescence from later stage trophozoites showed signal throughout the cytoplasm, with bright concentrations near or associated with the host erythrocyte membrane. These observations are consistent with transient association of KAHRP at Maurer's clefts before the protein docks beneath the host erythrocyte membrane.
